# CT-Guided Percutaneous Cryoablation of a Renal Metastasis From Esophageal Adenocarcinoma

**DOI:** 10.7759/cureus.98808

**Published:** 2025-12-09

**Authors:** David Mina, Scotty Mooney, Elias Salloum, Hakob Kocharyan, Mustafa Al-Roubaie

**Affiliations:** 1 Interventional Radiology, Moffitt Cancer Center, Tampa, USA; 2 Interventional Radiology, University of South Florida Morsani College of Medicine, Tampa, USA

**Keywords:** cryoablation, ct guided ablation, esophageal adenocarcinoma, image guided therapy, interventional radiology, oligometastasis, percutaneous ablation, renal metastasis, thermal ablation, tumor ablation

## Abstract

Renal involvement in esophageal adenocarcinoma is an uncommon clinical finding. We describe the case of a 74-year-old man with stage IV esophageal adenocarcinoma and prior hepatic, retroperitoneal nodal, and cerebral metastases who developed a new hypermetabolic lesion in the left kidney on Positron Emission Tomography/Computed Tomography (PET/CT). MRI identified a 3.4 cm enhancing mass, and biopsy confirmed poorly differentiated metastatic adenocarcinoma. The lesion was treated with CT-guided cryoablation using a dual freeze-thaw protocol. The patient tolerated the procedure well, and a small upper-pole bleed identified immediately post-procedure was successfully managed with selective coil embolization. He resumed systemic therapy and remained asymptomatic at the four-week follow-up, with a complete metabolic response at the ablation site on the follow-up PET/CT. This case illustrates the feasibility of CT-guided percutaneous cryoablation as a minimally invasive option for local control of renal metastases in patients with esophageal cancer.

## Introduction

Esophageal adenocarcinoma most commonly metastasizes to the liver, lungs, bone, and lymph nodes, while renal involvement is uncommon and primarily reported in isolated case reports or small series [1,2]. These reports suggest that renal metastasis occurs far less frequently than the involvement of typical metastatic sites, and the true incidence remains undefined [1,2]. Because such presentations are infrequent, management strategies for isolated renal metastases remain undefined [1,2].

Image-guided percutaneous ablation offers a minimally invasive means of achieving local tumor control while preserving renal function and minimizing interruption of systemic therapy [3-6]. Among the available modalities, cryoablation provides precise visualization of the ablation zone and limits collateral injury [4-6]. Its ability to monitor the ice-ball margin in real time, preserve surrounding renal parenchyma, and offer nephron-sparing treatment makes it particularly relevant for managing uncommon, isolated renal lesions in patients who may not be ideal surgical candidates [3,5].

This report describes a case of computer tomography (CT)-guided percutaneous cryoablation for a renal metastasis of esophageal adenocarcinoma, demonstrating the feasibility and safety of image-guided therapy in an uncommon metastatic setting.

## Case presentation

**Figure 1:**
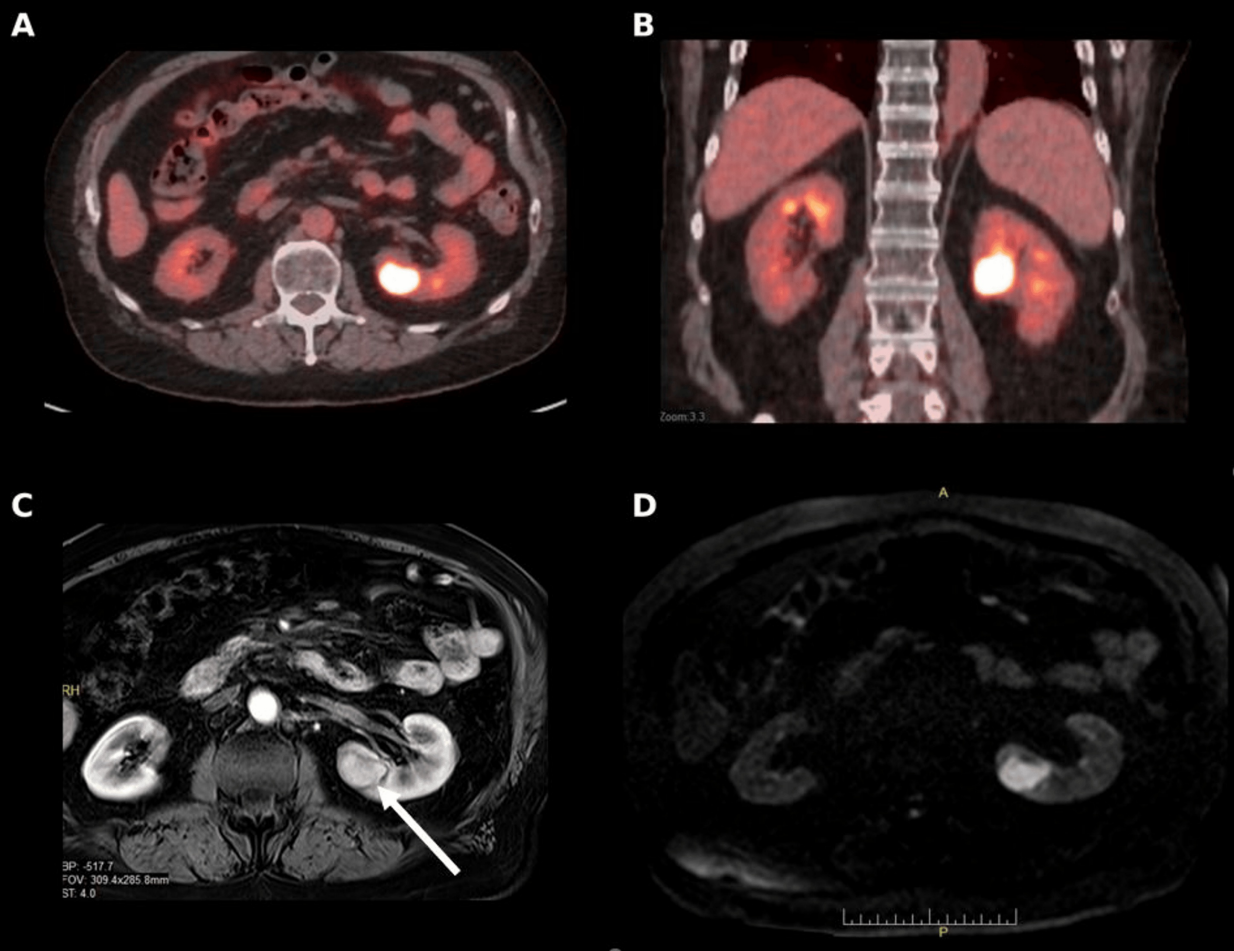
PET/CT and MRI demonstrating hypermetabolic left renal lesion (A) Axial positron emission tomography/computed tomography (PET/CT) demonstrating intense fluorodeoxyglucose (FDG) uptake in the mid-upper pole of the left kidney; (B) Coronal PET/CT confirming the hypermetabolic focus; (C) Contrast-enhanced T1-weighted magnetic resonance imaging (MRI) showing a 3.4 cm enhancing mass in the same region (white arrow); (D) Diffusion-weighted MRI demonstrating restricted diffusion at the lesion site, consistent with metastatic disease.

A 74-year-old man with stage IV esophageal adenocarcinoma and known metastases to the liver and retroperitoneal lymph nodes was under active systemic therapy. He had previously undergone stereotactic body radiation therapy to the retroperitoneal lymph nodes and craniotomy followed by postoperative radiotherapy for a left parietal brain metastasis. Routine surveillance Positron Emission Tomography (PET)/CT in July 2025 revealed a new hypermetabolic focus in the left kidney (Figure 1).

Contrast-enhanced magnetic resonance imaging (MRI) demonstrated a 3.4 cm solid enhancing mass in the mid-upper pole of the left kidney (Figure 1). At the time of detection, the patient had no renal-related symptoms, and baseline renal function was normal (estimated glomerular filtration rate >60 mL/min/1.73 m²). CT-guided biopsy confirmed poorly differentiated carcinoma consistent with metastatic esophageal adenocarcinoma.

**Figure 2:**
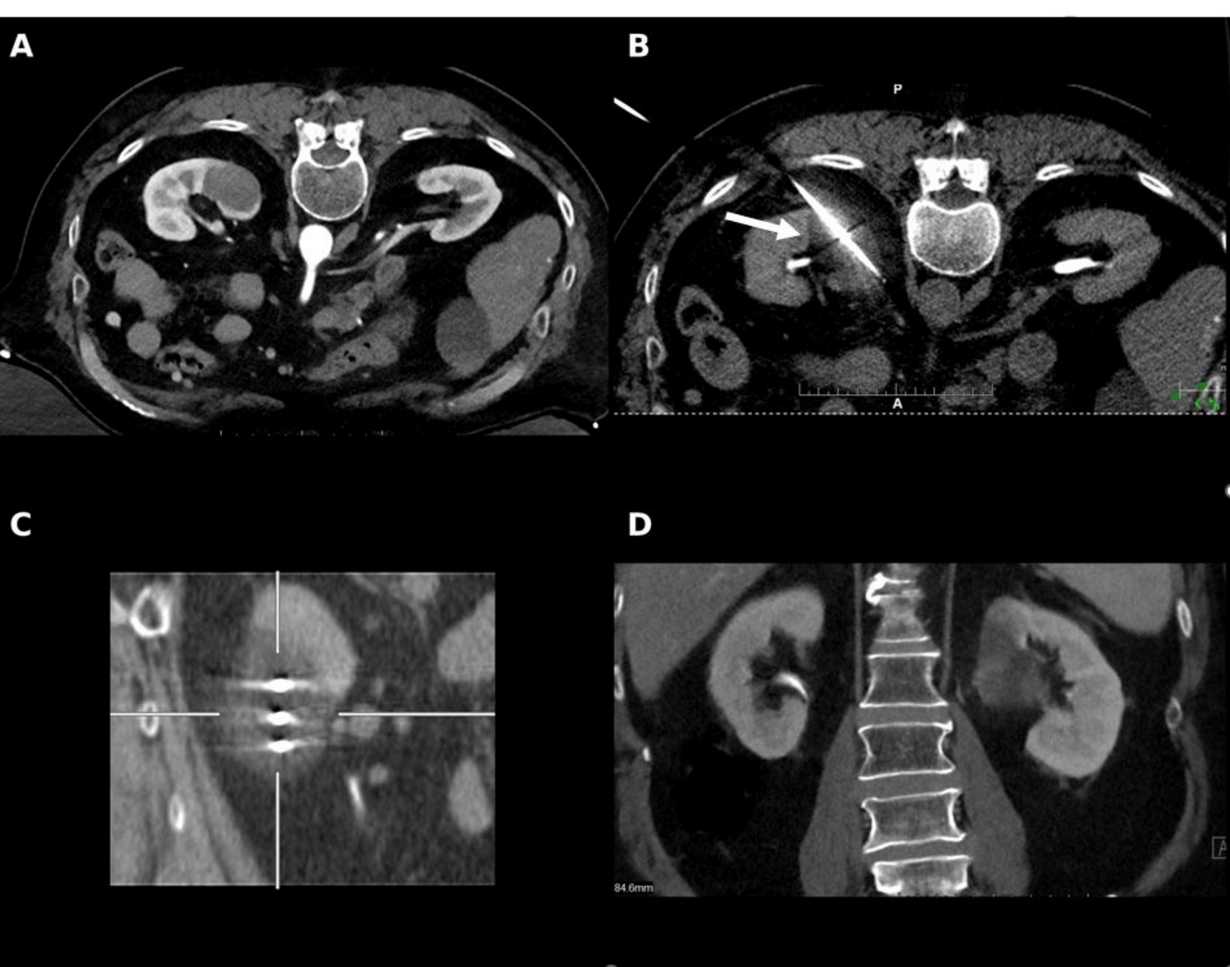
CT-guided percutaneous cryoablation of the left renal metastasis (A) Pre-ablation contrast-enhanced axial CT image used for trajectory planning and localization of the left renal lesion; (B) Intraprocedural axial CT showing cryoprobe placement and developing ice-ball (white arrow) encompassing the lesion; (C) Sagittal intraprocedural CT demonstrating all three cryoprobes within the tumor and the extent of ice-ball formation; (D) Coronal post-ablation CT demonstrating complete coverage of the lesion, consistent with successful ablation.

After a multidisciplinary tumor board discussion, percutaneous cryoablation was selected to achieve local disease control while allowing uninterrupted systemic therapy. The procedure was performed under general anesthesia with the patient in a prone position. A radiopaque skin grid and limited abdominal CT were used for localization, and contrast-enhanced CT was obtained to delineate lesion borders and guide probe trajectories (Figure 2).

Three Boston Scientific cryoprobes were placed under CT guidance-two IceForce 2.1 CX (14-gauge) probes (Boston Scientific Corporation, Massachusetts, USA) in the superior and mid-portions of the lesion and one IceRod 1.5 CX (17-gauge) probe inferiorly (Figure 2). A dual freeze-thaw protocol was used consisting of a 10-minute freeze, an eight-minute thaw, and a second 12-minute freeze. Intermittent CT imaging confirmed accurate probe positioning and complete ice-ball coverage with a five-millimeter safety margin (Figure 2). The procedure lasted approximately 90 minutes.

**Figure 3:**
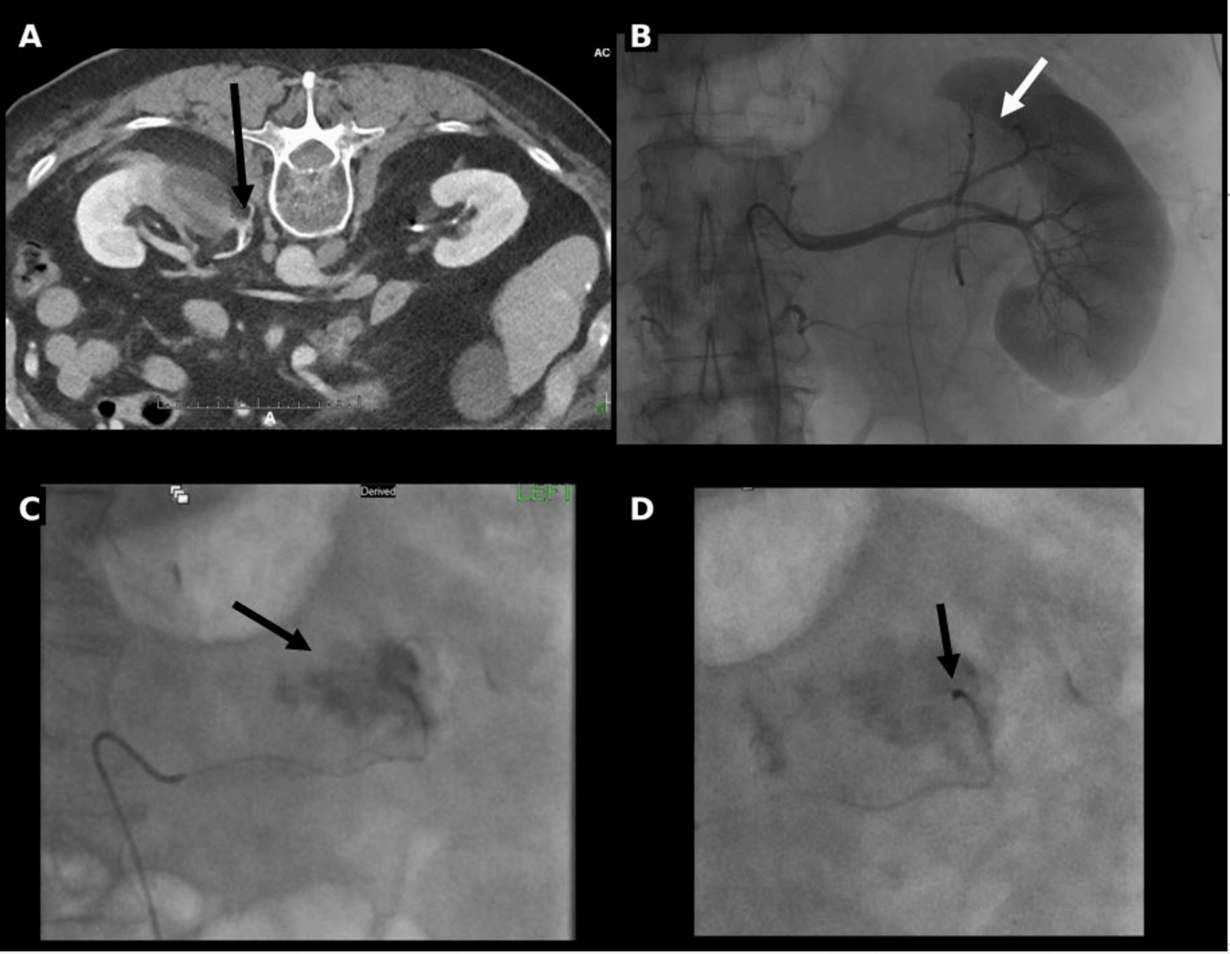
Angiographic evaluation and coil embolization of the post-ablation hemorrhage (A) Axial contrast-enhanced CT image obtained post-ablation, demonstrating a small perinephric hematoma and active extravasation (black arrow); (B) Selective left renal arteriogram demonstrating the ablation zone with the corresponding area of hypoenhancement (white arrow); (C) Superselective angiogram showing contrast extravasation from a distal upper pole segmental artery branch (black arrow); (D) Post-embolization angiogram following deployment of a 2 mm detachable coil (black arrow) within the culprit vessel, resulting in occlusion and resolution of the extravasation.

Post-procedural contrast-enhanced CT demonstrated a small perinephric contrast extravasation suggesting a small upper pole bleed (Figure 3).

**Figure 4:**
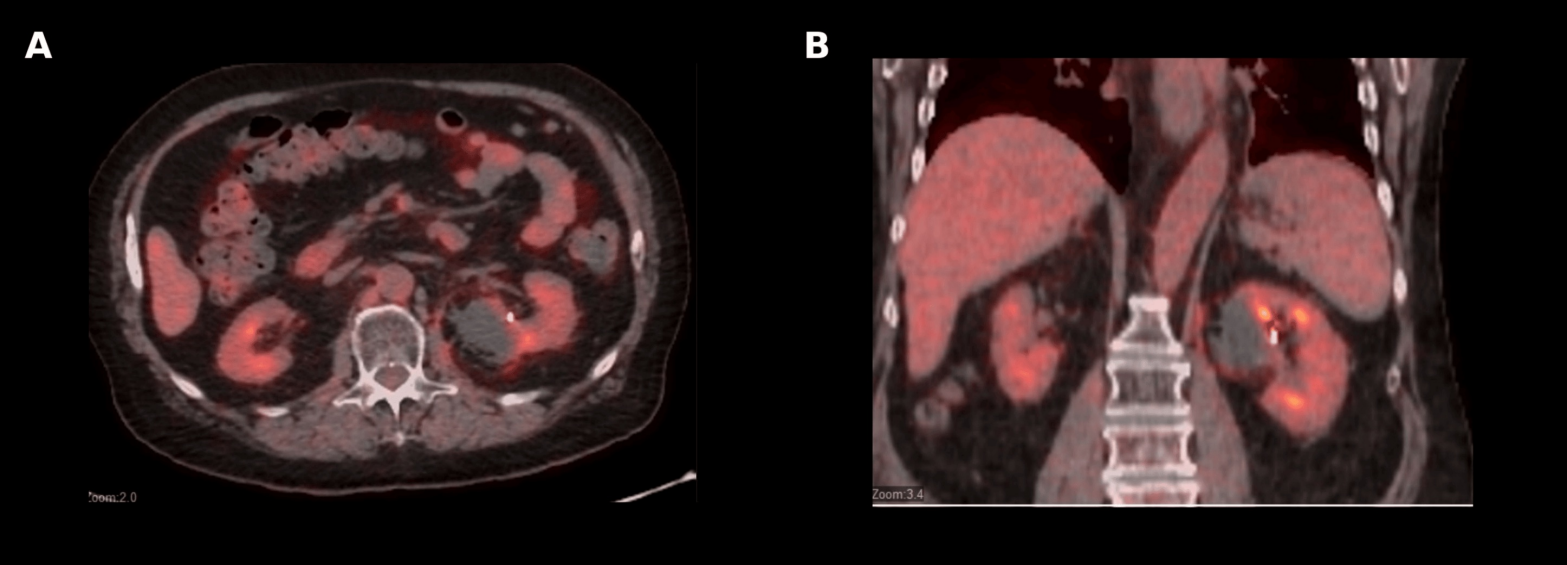
Four-week post-ablation PET/CT demonstrating interval metabolic response (A) Axial positron emission tomography/computed tomography (PET/CT) image obtained four weeks after ablation showing no abnormal fluorodeoxyglucose (FDG) uptake at the ablation site in the left kidney; (B) Coronal PET/CT image demonstrating an absence of residual or recurrent metabolic activity within the treated region, consistent with a favorable post-treatment response.

Diagnostic angiography confirmed bleeding from a distal upper pole segmental branch of the left renal artery, which was successfully treated with selective coil embolization (Figure 3). Despite the small post ablation bleed, blood loss remained minimal (estimated <50 mL), with no significant change in hemoglobin following the procedure. The patient recovered uneventfully and was discharged the next day. At the four-week follow-up visit, he remained asymptomatic without flank pain or hematuria, and his Eastern Cooperative Oncology Group performance status was zero. PET/CT performed at that time demonstrated expected post-ablation and embolization changes with an absence of metabolic activity within the lesion (Figure 4).

Renal function was unchanged post ablation with maintained glomerular filtration rate above 60 mL/min/1.73 m². 

## Discussion

Renal parenchymal metastasis from esophageal carcinoma is exceptionally uncommon and is more frequently observed in squamous cell histology than adenocarcinoma. Most reported cases are discovered incidentally during imaging surveillance or autopsy. Because these lesions often mimic primary renal neoplasms radiographically, image-guided biopsy remains essential for establishing the diagnosis and guiding appropriate management [1,2].

Role of image-guided cryoablation

Percutaneous image-guided cryoablation is a well-established therapy for primary renal tumors and has increasingly been utilized for select renal metastases [3,4]. Advantages over other thermal ablation modalities include direct visualization of the ice ball, real-time monitoring of the ablation margins, and reduced risk of collateral damage to the adjacent structures [5]. In the present case, cryoablation enabled precise targeting and coverage of a biopsy-proven metastatic lesion while preserving the renal parenchyma and avoiding interruption of systemic therapy.

Complication recognition and management

The occurrence of a small upper pole bleed immediately post-procedure underscores the importance of prompt recognition and management of vascular complications. Diagnostic angiography allowed identification of the bleeding source and successful treatment with selective coil embolization, achieving complete hemostasis and preservation of renal function. This highlights the advantage of performing percutaneous ablation in an interventional radiology suite where immediate angiographic management is available [6].

Clinical implications and novelty

To our knowledge, no prior cases of percutaneous cryoablation for a renal metastasis of esophageal adenocarcinoma have been reported in the literature. This case expands the clinical application of image-guided ablation to a previously undocumented metastatic setting. In patients with otherwise stable systemic disease, local control of isolated metastases can prolong the effectiveness of systemic therapy and maintain quality of life. The favorable outcome in this case demonstrates that percutaneous cryoablation can provide safe, effective local control of rare metastatic sites within a multidisciplinary treatment paradigm [7,8].

## Conclusions

CT-guided percutaneous cryoablation is a safe and effective technique for achieving local disease control in cases of a renal metastasis from esophageal adenocarcinoma. This case demonstrates that cryoablation can be successfully used beyond its well-established indications for primary renal neoplasms to treat rare metastatic deposits while preserving renal function and minimizing morbidity. The ability to perform precise, image-guided ablation allows patients to continue systemic therapy without interruption, maintaining both oncologic control and quality of life.

The present case also underscores the importance of multidisciplinary collaboration in managing complex metastatic disease. Early involvement of interventional radiology can offer minimally invasive alternatives to surgery, especially in patients with limited organ reserve or widespread disease. As image-guided therapies continue to evolve, their integration into comprehensive oncologic care pathways will likely expand, providing durable local control for selected patients with atypical metastatic patterns. Further documentation of similar cases, along with longer-term follow-up, will be essential to refine patient selection and establish procedural standards for these uncommon but clinically significant scenarios.

## References

[1] Kato H, Nakajima M (2013). Treatments for esophageal cancer: a review. Gen Thorac Cardiovasc Surg.

[2] Chang KP, Huang CP, Chang H (2016). Solitary renal metastasis of esophageal squamous cell carcinoma mimicking primary renal neoplasm - a case report and literature review. Biomedicine (Taipei).

[3] Georgiades CS, Hong K, Bizzell C, Geschwind JF, Rodriguez R (2008). Safety and efficacy of CT-guided percutaneous cryoablation for renal cell carcinoma. J Vasc Interv Radiol.

[4] Atwell TD, Schmit GD, Boorjian SA (2013). Percutaneous ablation of renal masses measuring 3.0 cm and smaller: comparative local control and complications after radiofrequency ablation and cryoablation. AJR Am J Roentgenol.

[5] Hinshaw JL, Lubner MG, Ziemlewicz TJ, Lee FT Jr, Brace CL (2014). Percutaneous tumor ablation tools: microwave, radiofrequency, or cryoablation-what should you use and why?. Radiographics.

[6] Ahmed M, Solbiati L, Brace CL (2014). Image-guided tumor ablation: standardization of terminology and reporting criteria-a 10-year update. Radiology.

[7] Rogers MP, DeSantis AJ, DuCoin CG (2021). Oligometastatic adenocarcinoma of the esophagus: current understanding, diagnosis, and therapeutic strategies. Cancers (Basel).

[8] Crocetti L, de Baere T, Lencioni R (2010). Quality improvement guidelines for radiofrequency ablation of liver tumours. Cardiovasc Intervent Radiol.

